# Enabling time resolved microscopy with random Raman lasing

**DOI:** 10.1038/srep44572

**Published:** 2017-03-15

**Authors:** Brett H. Hokr, Jonathan V. Thompson, Joel N. Bixler, Dawson T. Nodurft, Gary D. Noojin, Brandon Redding, Robert J. Thomas, Hui Cao, Benjamin A. Rockwell, Marlan O. Scully, Vladislav V. Yakovlev

**Affiliations:** 1 Texas A&M University, College Station, TX 77843 USA; 2 Engility, Joint Base San Antonio, Fort Sam Houston, TX 78227, USA; 3 711th Human Performance Wing, Human Effectiveness Directorate, Bioeffects Division, Optical Radiation Branch, Joint Base San Antonio, Fort Sam Houston, TX 78234, USA; 4 Yale University, New Haven, CT 06520, USA; 5 Princeton University, Princeton, NJ 08540, USA; 6 Baylor University, Waco, TX 76706, USA

## Abstract

Optical imaging of fast events and processes is essential for understanding dynamics of complex systems. A bright flash of illuminating light is required to acquire sufficient number of photons for superior image quality. Laser pulses can provide extreme brightness and are typically employed to achieve high temporal resolution; however, the high degree of coherence associated with the lasing process degrades the image quality with speckle formation. Random lasers are low-coherence sources of stimulated emission and do not suffer from speckle, but are rather broadband and have a relatively low output power limiting the scope of their potential applications. In this report, we demonstrate the use of random Raman lasing as a novel imaging light source with unprecedented brightness for a speckle-free and narrowband light source. We showcase the advantages of a random Raman laser to image the nanosecond scale dynamics of cavitation formation in water and quantitatively compare these images to those taken with incoherent fluorescent emission and coherent laser light as illumination source.

New imaging technology pulls science forward by opening a window to visualize unknown effects through novel modalities or increased resolution in space or time. The ability to image with higher resolution^1^,^2^ and higher speeds^3^ allows us to directly visualize phenomena, often times elucidating unexpected intricacies, and expanding our knowledge of the relevant processes. In wide-field microscopy, where the light source illuminates the entire field of view and the full image is acquired simultaneously, the speed at which an image can be acquired is limited by the amount of light that reaches the camera. In practice, this is limited by the brightness of the light source because most spatially incoherent light sources are thermal sources, relying on spontaneous emission. However, random Raman lasing^4^ works through a fundamentally different mechanism, allowing it to produce incoherent light that is effectively 10,000 times more bright than commonly used incoherent light sources. In this work, we will outline the fundamental properties of this light source which make it both a unique and powerful new tool, and demonstrate proof of principle images of shockwave formation in laser induced breakdown.

Traditional light sources for wide-field imaging are derived through spontaneous processes, such as spontaneous emission. Such sources, like mercury arc lamps, cannot be rapidly pulsed, and are typically limited in brightness by the heat generated through these incoherent mechanisms. On the other hand, lasers are generated in a highly coherent fashion and can produce incredibly bright and fast pulses of light; however, lasers are poorly suited for wide-field imaging due to their high degree of spatial coherence. In the presence of even small amounts of scattering, the coherence results in self-interference of the beam and leads to the formation of speckle patterns that significantly degrade the image.

Random lasers, or other highly multi-mode lasers, offer the unique combination of laser-like brightness, but extremely low coherence^5^,^6^,^7^,^8^. This makes them ideal sources for imaging applications^9^,^10^; however, their brightness tends to be limited due to reabsorption of the laser radiation and strong absorption of the pump laser, leading to damage to the lasing medium. Random Raman lasers^4^,^11^,^12^, where stimulated Raman scattering (SRS) is used as the gain mechanism, are a new source with some unique features that make them an attractive imaging source in their own right^13^. Random Raman lasers have the fundamental advantages that the lasing medium does not need to be highly absorbing at the pump wavelength and the emitted radiation is not resonant with any energy levels. Low absorption at the pump wavelength allows significantly more powerful pump laser pulses to be used, resulting in a much brighter emission. These pulses have been previously shown to be bright enough to observe from kilometers away^14^. The bright emission is illustrated schematically in Fig. 1. The overall lack of absorption at both the pump and emission wavelengths makes thermal management of the source entirely unnecessary as there is virtually no heating. Furthermore, random Raman lasers have a very narrow bandwidth, 8 cm^−1^ is typical (see Fig. 2). This unique property opens the door to potentially new imaging techniques, such as wide-field Raman microscopy, because a bright, narrowband, and speckle-free light source does not presently exist. Figure 1 quantifies this statement. When compared to other low-coherence light sources, random Raman lasing emission is orders of magnitude brighter in terms of the power useful to imaging. It is 10,000 times brighter than the commonly used mercury arc lamp for a process with an excitation bandwidth of about 10 nm, such as fluorescence microscopy. The random Raman laser used in this work had a pulse duration on the order of 1 ns, as measured by a streak camera (Hamamatsu). Therefore, this increased brightness directly allows temporal gating of faster dynamics. Additionally, weaker contrast modalities like Raman spectroscopy become feasible for wide-field imaging.

Here we will demonstrate proof of principle results using random Raman lasing emission as a strobe light source to illustrate the nanosecond dynamics of laser induced breakdown in water. Understanding the mechanical effects of laser induced breakdown in water has important consequences in laser surgery^15^. Often times, the shock-wave resulting from the breakdown leads to secondary damage and reduces the resolution of the procedure^16^. Additionally, we will discuss the spatial coherence and spectral properties of the random Raman lasing emission that make it a completely unique light source and compare the quality of images obtained to other light sources.

## Methods

**Figure 2:**
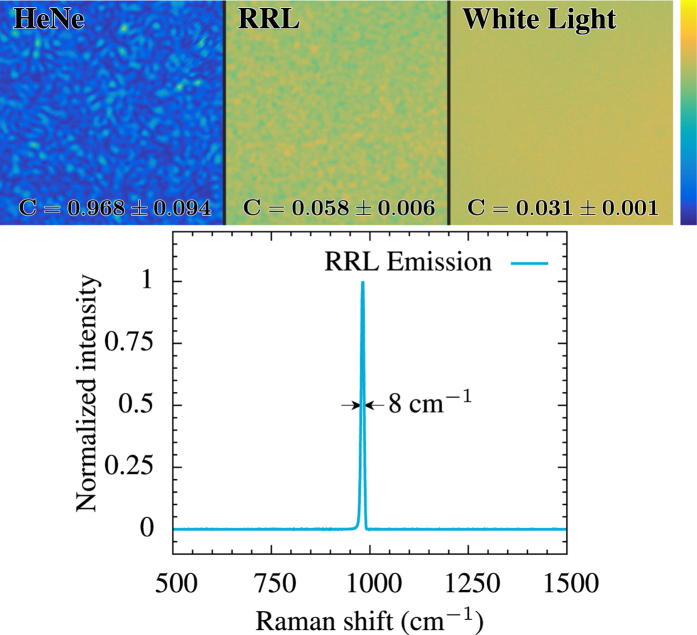
(Top) Speckle contrast of random Raman laser (RRL) emission compared to highly coherent and highly incoherent sources. (Bottom) Emission spectrum of the random Raman laser. This wavelength shift corresponds to 562 nm emission when pumped with a frequency doubled Nd:YAG laser at 532 nm. At this pumping frequency, this emission width corresponds to a 0.25 nm spectral width, making it exceptionally narrowband compared to other spatially incoherent sources.

To demonstrate the capability of random Raman lasing as an imaging strobe, the speckle contrast of the light was measured. To do this, we first passed the light through a 2 m long 600 *μ*m fiber. The light from the fiber was left uncollimated and allowed to overfill the array of a 16-bit CCD (Orca-ER; Hamamatsu). The CCD was placed sufficiently far from the fiber so that the speckle grain size was significantly larger than the pixel size of the CCD. To demonstrate the capabilities of the setup and provide a basis to compare the random Raman lasing emission we used a helium neon laser as a highly coherent source, and a halogen light as a low-coherence source. The resulting images and speckle contrast are shown in Fig. 2. To measure the speckle contrast we have chosen a 400 × 400 region of the CCD that was clear of debris and subdivided that into 25 80 × 80 sub-regions. The speckle contrast of each sub region is computed independently by first subtracting a pedestal to reduce the effect of the CCD dark counts on the calculation then computing the average and standard deviation^17^,^18^. This helps minimize any effects due to non-uniform illumination of the array. Multiple images were taken. Those used in the calculations for the random Raman lasing and halogen light source were selected by rejecting any images with saturated pixels and requiring the average counts per pixel to be greater than 20,000. This minimizes the effect of noise due to the CCD while ensuring that saturation is not occurring. For the Helium-Neon (HeNe) images, the requirement on average pixel count was not enforced, but the laser power was adjusted to maximize the dynamic range of the detector without saturation. All together, 21 images were used for the HeNe calculations, 28 images for the random Raman lasing emission, and 101 images for the white light. All 25 sub-regions from each image were pooled together and averaged. The errors quoted in Fig. 2 are the standard deviation of these averages.

**Figure 3:**
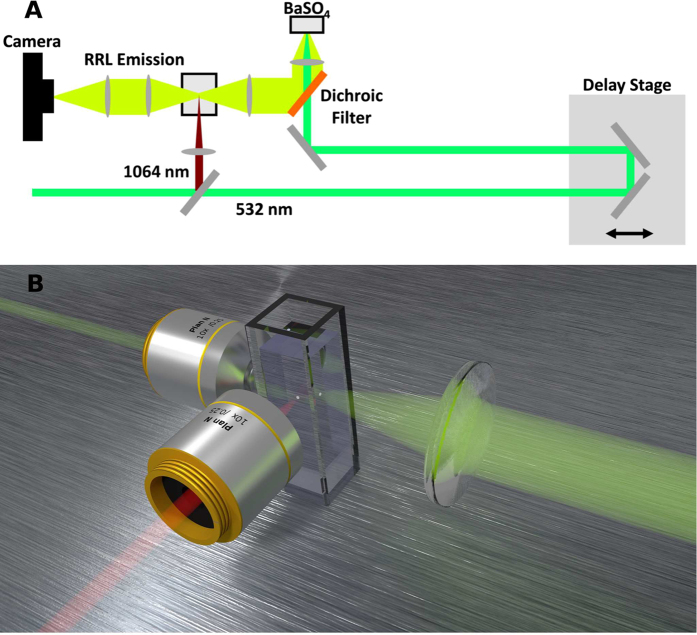
Schematic diagram of the experimental setup showing the yellow-green random Raman laser emission being used to strobe the breakdown produced by the 1064 nm laser pulse (illustrated in red).

To image laser induced breakdown in water the setup pictured schematically in Fig. 3 was used. A picosecond seeded Nd:YAG regenerative amplifier (Quanta-Ray GCR-3RA; Spectra Physics) generated 50-ps pulses at a repetition rate of 10-Hz. The residual 1064-nm light from the doubling process was split off and focused into a cuvette containing water using a 10x long working distance microscope objective (Mitutoyo). The power of the 1064-nm laser was adjusted using neutral density filters to get a pulse energy of 70 uJ. The 532-nm pulse out of the laser was sent through a delay stage and reflected off a longpass dichroic beamsplitter (Di02-R532; Semrock) prior to being focused onto barium sulfate powder (ReagentPlus; Sigma-Aldrich) that was lightly packed into a 1-cm diameter, 1-cm deep container. Before being focused onto the powder, the 532-nm pulse energy was 1.7 mJ. The sample was placed in front of the focal plane so that the beam on the surface of the powder was approximately 1-mm in diameter. The resulting random Raman lasing emission was transmitted through the dichroic beam-splitter and residual 532-nm light was further attenuated by an additional 6 OD notch filter (NF533-17; ThorLabs). The random Raman laser emission was then used to illuminate the laser induced breakdown as shown in Fig. 3B. The laser induced breakdown was imaged to a CCD (Orca-100; Hamamatsu) using an identical 10x microscope objective and a 500 mm focal length lens for a total magnification of 25x. A 560 nm bandpass filter was used to reject any plasma emission or higher order random Raman emission lines^19^.

## Results and Discussion

The spatial coherence of the emission can be quantified through the speckle contrast, *C* = *σ*/〈*I*〉 where 〈*I*〉 is the average value of the intensity and σ is the standard deviation^20^,^21^. This allows a quantitative comparison of the spatial coherence of any source. As illustrated in Fig. 2, a small amount of spatial coherence persists in random Raman lasing. In practice, this is an acceptable amount of speckle for the vast majority of imaging applications as it is considerably lower than that of other commonly used sources, such as superluminescent diodes^9^. Furthermore, the random Raman laser has been observed to generate a unique speckle pattern with each laser pulse, allowing the speckle contrast to be further reduced by averaging over multiple pulses if the application allows.

**Figure 4:**
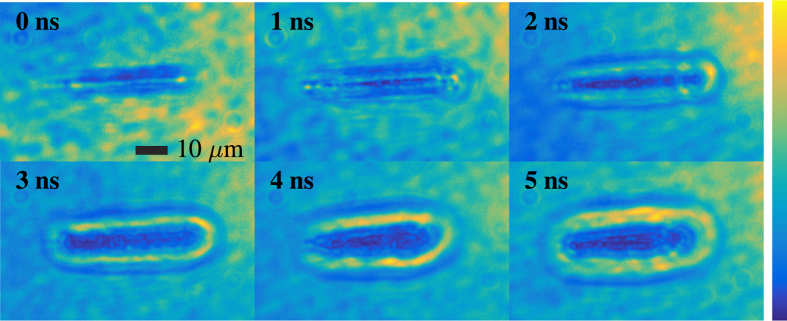
Images of laser induced breakdown in water using random Raman lasing emission as the strobe. The images have been scaled to maximize the contrast, but no other image processing was done.

The acquired images of laser induced breakdown illuminated by random Raman laser emission are shown in Fig. 4. A delay stage was used to delay the random Raman laser pump pulse to acquire images at different delay times. Time zero corresponds to when the breakdown pulse and random Raman laser strobe pulse arrive at the same time. The images in Fig. 4 were scaled to maximize the contrast but no further image processing was done on these images. The resulting shock wave can be clearly seen moving out from the initial breakdown event as time progresses in addition to small-scale details showing the inhomogeneous nature of the breakdown event. There is slight non-uniformity in the illumination, but this could easily be corrected for through background subtraction or passing the illumination through a multi-mode fiber and imaging the tip of the fiber through the system.

**Figure 5:**
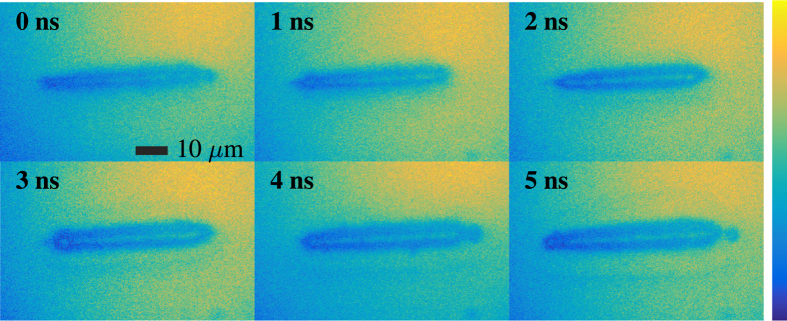
Images of laser induced breakdown in water using the fluorescence from Rhodamine 590 dye pumped by the 50 ps 532 nm laser pulse as the strobe. The images have been scaled to maximize the contrast, but no other image processing was done.

**Figure 6:**
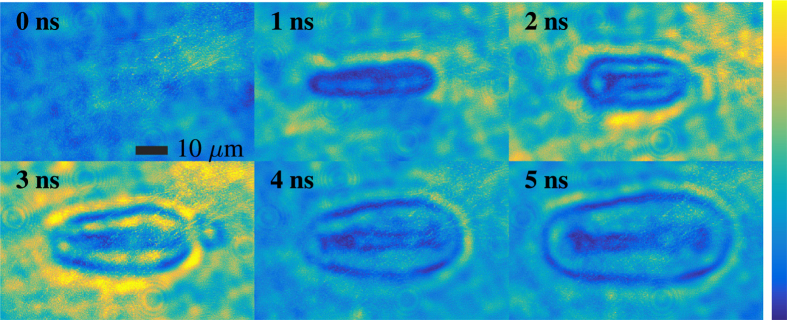
Images of laser induced breakdown in water using the 50 ps 532 nm pulse from the laser as the strobe. The images have been scaled to maximize the contrast, but no other image processing was done.

In an attempt to obtain a fair comparison of the quality of images obtained using random Raman laser emission, we repeated the same experiment using two other light sources. First, we used the fluorescence emission from a Rhodamine 590 dye pumped with the same 532 nm laser pulse used to generate the random Raman laser. These results are shown in Fig. 5. The most apparent shortcoming in these images is the bad temporal resolution resulting from the relatively long fluorescence lifetime (several nanoseconds^22^) of the dye on these timescales. The poor temporal resolution results in nearly a complete loss in the fine features visible under random Raman laser emission. Next, we utilized the green 532 nm pulse from the laser itself as the strobe (see Fig. 6). The laser provides superior temporal resolution, however, the spatial coherence of the source can degrade the image through speckle formation, even though there is very little scattering present in the system. As a result of this speckle degradation, the small details in the center of the breakdown are poorly resolved due to the small amount of speckle present. This would be greatly exasperated in the presence of scattering, as would be the case in any biological sample.

**Figure 7:**
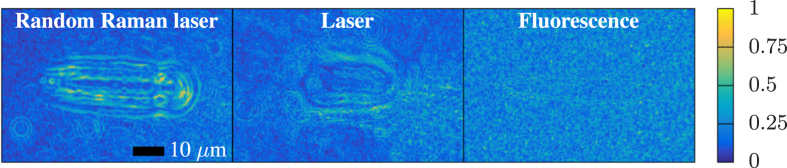
Images from Figs 3, 4 and 5 at 2 ns delay passed through a Sobel filter to compute the gradient. The magnitude of the gradient is shown. This kind of filter is commonly used in image processing for edge detection as it highlights areas where contrast is high. Thus, in this case it is being used to highlight the higher degree of detail captured by random Raman lasing due to the lack of speckle while maintaining sufficient temporal resolution to capture the laser induced breakdown event.

**Figure 8:**
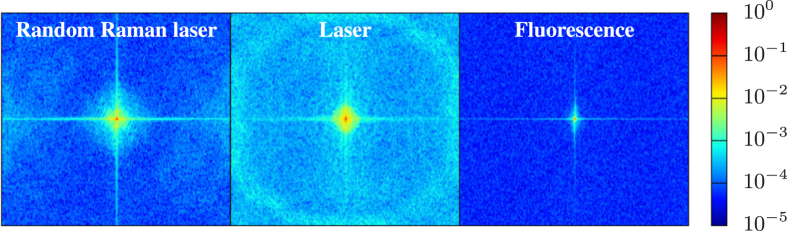
Fast Fourier transform of images from Figs 3, 4 and 5 at 2 ns delay illustrating the spatial frequency spectrum of the images. The narrow spike in the fluorescence spectrum is the result of the lack of sharp details in the image, and the broad spectral noise background in the laser image is the result of background noise due to the formation of laser speckle. In contrast the random Raman laser image demonstrates a broad range of spatial frequencies, allowing for the resolution of fine details, while maintaining a much smaller background due to speckle formation.

The quality of these images is a difficult metric to scientifically quantify; however, it is possible to look at measures of the images that illustrate the spatial frequencies present as well as the sharpness of the images. We applied a Sobel filter, which computes the gradient of the image, to the images taken with each light source. The results very clearly highlight the resolution of small-scale features in the center of the breakdown under random Raman laser illumination that are not well resolved by the laser and are totally non-existent with the fluorescence source. These results are highlighted in Fig. 7. Another measure of the detail captured in an image is the amount of spatial frequencies present. To assess this we applied fast Fourier transforms to the same raw images (results shown in Fig. 8). We find that the fluorescence images support only low spatial frequencies, consistent with the temporal smearing present, and while the laser supports significantly higher spatial frequencies in the image, it has a large high frequency background due to speckle. In comparison, the images acquired with random Raman laser illumination shows the presence of high spatial frequencies, but with a greatly reduced high frequency background compared to the laser illumination.

In addition to exhibiting exceptional brightness and nearly speckle-free behavior, random Raman laser emission has the advantage that the wavelength can be changed relatively simply. In our present setup, the emission undergoes a 985 cm^−1^ Raman shift from the 532 nm pump light due to barium sulfate (BaSO_4_), resulting in emission at 562 nm. However, random Raman lasing is capable of producing bright higher-order effects that lead to additional emission at 595 nm, and even 632 nm^19^. These higher-order emission lines have been shown to be remarkably efficient. Furthermore, they would offer an opportunity for nanosecond scale multispectral imaging from a single light source simultaneously through the use of simple band pass filters to select which emission line goes to which camera. In addition to offering wavelength selectability through higher-order emission lines, the emission wavelength can be tuned by changing the wavelength of the pump laser. This could have tremendous applications to fluorescence microscopy where the illumination pulse could be optimized to maximize the quantum efficiency of the fluorescent molecule of interest.

## Conclusion

Random Raman lasing emission has the capability to provide very bright, narrow-band, and low spatial coherence light source that can be used for full-field microscopy. When compared to other low-coherence light sources, the advantage is clear. The random Raman laser is capable of delivering 10,000 times the useful peak power of mercury arc lamps for a technique such as fluorescence imaging while not suffering from the laser speckle present when laser sources are used. This will open up a window to study faster dynamics and potentially new applications using weaker, but more specific modalities such as Raman spectroscopy. Finally, we demonstrate proof of principle images of laser induced breakdown in water and demonstrate that the images illuminated under random Raman laser strobes contain more detailed features than those illuminated with a laser strobe or a fluorescence strobe. Additionally, these proof of principle results provide direct evidence that random Raman laser emission can produce wide-field images with nanosecond temporal resolution with minimal image degradation due to spatial coherence effects.

## Additional Information

**How to cite this article:** Hokr, B. H. *et al*. Enabling time resolved microscopy with random Raman lasing. *Sci. Rep.* **7**, 44572; doi: 10.1038/srep44572 (2017).

**Publisher's note:** Springer Nature remains neutral with regard to jurisdictional claims in published maps and institutional affiliations.

## Figures and Tables

**Figure 1:**
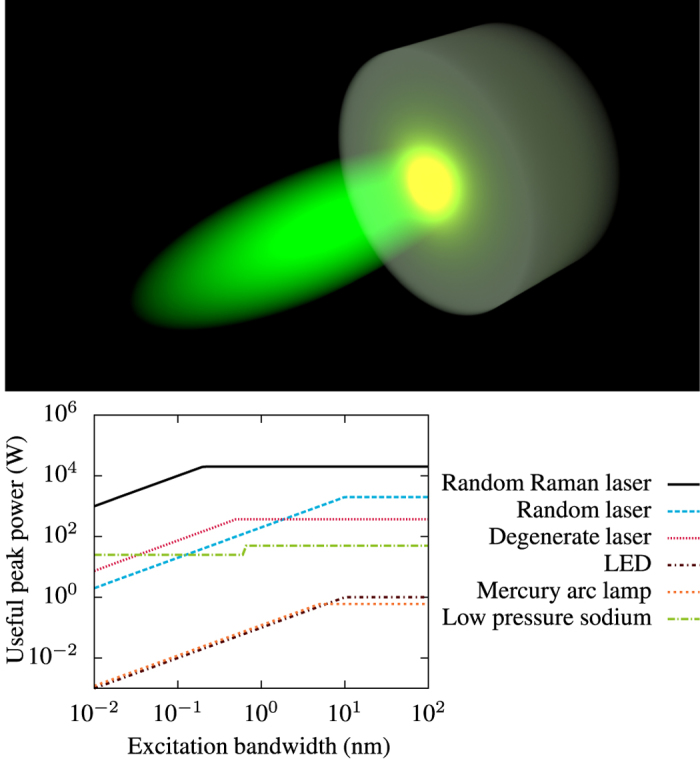
(Top) Conceptual image of the random Raman laser emission (yellow) being stimulated by the incident pump pulse (green). Here it is depicted that while the pump pulse is directional, the emitted random Raman laser light is non-directional. (Bottom) Comparison of the useful brightness of various low-spatial coherence light sources compared to the excitation bandwidth of the imaging process. For example, typical fluorescence imaging has a characteristic excitation bandwidth on the order of 10 nm. Useful peak power is defined as the peak power achieved in the specified bandwidth at the optimum wavelength for that light source. In this way, useful peak power is a measure of how much light is absorbed by the sample which is then multiplied by the quantum efficiency of the process to obtain the peak power observed by the camera. The degenerate laser is described in Nixon *et al*.^5^. Other values were estimated from the specifications of commercially available sources.
